# Mechanisms of dark personality traits in lesbian adult attachment: a chain-mediated model

**DOI:** 10.3389/fpsyg.2025.1619432

**Published:** 2025-07-23

**Authors:** Ye Liu, Shujiao Li, Shaoyang Ma

**Affiliations:** ^1^School of Psychology and Mental Health, North China University of Science and Technology, Tangshan, China; ^2^Hebei Key Laboratory of Mental Health and Brain Science, Tangshan, China

**Keywords:** dark personality traits, adult attachment, internalised homophobia, self-esteem, lesbian

## Abstract

**Introduction:**

Recent years have witnessed a growing focus on the mental health of sexual minorities, however research on the adult attachment styles of lesbians remains limited. This study examined the impact of dark personality traits on adult attachment, as well as the mediating roles of internalised homophobia and self-esteem among lesbian women.

**Methods:**

A total of 1136 lesbians (*M_age_* = 21.57, *SD_age_* = 3.716) were surveyed using the Dirty Dozen, the Experiences in Close Relationships Inventory, the Internalised Homophobia Scale, and the Rosenberg Self-Esteem Scale. With dark personality traits as the independent variable, internalised homophobia and self-esteem as the mediating variables, and the two dimensions of adult attachment as the dependent variables, the relationship among these the variables was tested using the PROCESS plug-in for SPSS version 27.0.

**Results:**

The results indicated that dark personality traits are strongly correlated with internalised homophobia and are a significant factor influencing it (*β* = 0.052, p < 0.001). Internalised homophobia, in turn, significantly negatively impacted self-esteem (*β* = −0.039, p < 0.001). Furthermore, self-esteem significantly negatively influenced both attachment avoidance (*β* = −1.033, p < 0.001) and attachment anxiety (*β* = −1.246, p < 0.001). These findings support the chain mediation hypothesis, revealing three distinct pathways: (1) dark personality traits → internalised homophobia → attachment avoidance, attachment anxiety, (2) dark personality traits → self-esteem → attachment avoidance, attachment anxiety, and (3) dark personality traits → internalised homophobia → self-esteem → attachment avoidance, attachment anxiety.

**Discussion:**

These results highlight the significant impact of dark personality traits on attachment patterns among lesbians, offering valuable insights for counselling and clinical practice tailored to lesbians.

## 1 Introduction

As society’s understanding of various sexual orientations deepens, research on the mental health of sexual minorities has garnered widespread attention in recent years (Amos et al., 2020; Chan et al., 2022; Horton, 2022; Parra et al., 2023; Abboud et al., 2025; Clare et al., 2024). The Minority Stress Mediation Model posits that, despite the decriminalization and depathologization of homosexuality in society (Drescher, 2015; Roberts, 2019; McHenry, 2022; Shalahuddin et al., 2023; Wajhat et al., 2024), many individuals in the general public continue to perceive homosexuality as a pathological behavior (Hall and Rodgers, 2019; Balaji et al., 2023; Lin and Lee, 2024; Wilson et al., 2024). This persistent stigma has resulted in a higher prevalence of psychological disorders among sexual minorities, with depression rates in this group reaching 26%, significantly higher than those found in the heterosexual population (O’Shea et al., 2025). Sexual minority women (42.9%) who have experienced discrimination are more likely to suffer from mood disorders and chronic anxiety disorders (Lee et al., 2016). Lesbianism, a significant category among sexual minorities, pertains to women who experience romantic and sexual attraction exclusively toward other women. Even with growing focus on LGBTQ+ matters, studies continue to emphasize the experiences of gay men, leading to a consistent oversight of the unique psychosocial dynamics faced by lesbians. Current research on lesbian communities mainly focuses on cultural portrayals, the negotiation of identity, and mental health (Yudhistira et al., 2016; Jones, 2018; Reddy-Best and Jones, 2020; Annati and Ramsey, 2022; Hang and Zhang, 2023; Valderrama-Burgos, 2024), while largely overlooking how personality constructs interact with attachment dynamics in shaping intimate partnerships. This gap is especially noticeable in China, where there is a lack of research on the psychological processes that contribute to the formation of attachment patterns in lesbian individuals. This deficiency hinders theoretical progress in understanding relationship development and the creation of evidence-based interventions for this group.

Dark personality traits, s which include machiavellianism, narcissism, and psychopathy, is characterized by low empathy, high manipulation, and antisocial behaviors (Zeigler-Hill and Vonk, 2015). It is often considered an antisocial personality trait in Western society (Schild et al., 2020; Aghababaei et al., 2022; Rabl et al., 2024; Pechorro et al., 2025). In heterosexual populations, research has demonstrated a notable link between dark personality traits and adult attachment styles (Brewer et al., 2018). However, studies by Hogan (2017) have also shown that dark personality traits can contribute to personal success. For instance, when high levels of these traits are combined with intelligence and physical beauty, individuals are more likely to attain leadership positions (Ahmed and Islam, 2023). Additionally, men who exhibit high levels of these traits are perceived as more attractive in intimate relationships (Larsen, 2024). In contrast, there is a notable absence of systematic exploration into the impact of dark personality traits on attachment patterns within the lesbian community, especially regarding how these traits interact with social pressures and self-identity. Therefore, this study aims to investigate the mechanisms through which dark personality traits affect lesbian attachment avoidance and attachment anxiety, emphasizing the mediating roles of internalised homophobia and self-esteem between these variables. The goal is to strengthen attachment styles within the lesbian community and boost their overall well-being in intimate relationships.

## 2 Hypothesis

### 2.1 Dark personality and adult attachment

Adult attachment refers to the stable and enduring emotional bonds that individuals form with their peers throughout their interactions (Karantzas et al., 2023). Attachment theory posits that the patterns of interactions between individuals and their caregivers during infancy are gradually internalised into mental representations that guide how they navigate intimate relationships, i.e., the ‘internal working model’ (Chen et al., 2020). Instability in the internal working model is a significant characteristic of insecure attachment (Wang et al., 2024). Individuals with high level of avoidance tend to prioritize control within the relationship and often employ strategies such as withdrawal and escape (Takayanagi et al., 2024), in contrast, individuals with high level of anxiety are more concerned about their safety and are likely to harbor negative perceptions of the intimate relationship, as well as hostile and rebellious intentions towards their partner (Stefania et al., 2023; Fernandes et al., 2016).

Dark personality traits is a novel cluster of characteristics that exist in the grey area between clinically pathological and healthy personality (Moshagen et al., 2018). Individuals with high levels of these traits are more likely to demonstrate insecure attachment styles, and the behavioral traits and relational styles embodied in this trait predict elevated levels of adult attachment (Brewer et al., 2018). Research has indicated that Machiavellianism is positively correlated with attachment avoidance within the gay male population (Madonna and Rangaiah, 2023). Furthermore, individuals with pronounced dark personality traits often struggle to establish a strong sense of trust with others, and interpersonal trust is significantly negatively correlated with adult attachment (Harrison et al., 2018). In lesbian populations, elevated dark personality traits may intensify internalised prejudice, resulting in greater psychological alienation, while their low empathy trait is more likely to provoke interpersonal conflict (Brazil et al., 2023). Therefore, the hypothesis 1 is proposed: Dark personality traits co-influences adult attachment in lesbians (H1).

### 2.2 The mediating role of internalised homophobia

Internalised homophobia refers to the negative attitudes that individuals may develop toward their own sexual orientation as a result of internalizing external prejudices. This phenomenon often aligns with mechanism of role selection (Lin et al., 2022). Numerous studies highlight that characteristics such as ‘exaggerated self-esteem, low empathy, and poor interpersonal sensitivity’ are clear signs of a dark personality, exemplified by narcissists who openly showcase themselves while neglecting the feelings of others (Huprich et al., 2016; Zuo et al., 2016; Rogoza et al., 2018). However, more recent research indicates that the underlying traits of narcissistic personality disorder include ‘unstable self-worth’ and ‘heightened sensitivity to social rejection’, collectively known as ‘narcissistic vulnerability’. This fragile form of narcissism is linked to internalization (Rogoza et al., 2022). Vulnerable narcissists struggle to maintain close relationships due to their ongoing need for validation and their defensive reactions to criticism, which hinder their social interactions (Lamkin et al., 2015). This challenge is particularly pronounced for homosexual individuals, who may find it hard to navigate social settings in the face of systemic prejudice, such as homophobia, leading to increased self-doubt and heightened defensiveness, emotional instability, and internalised self-hatred.

Research has demonstrated that internalised homophobia can indirectly heighten attachment anxiety through feelings of loneliness (Hu J. C. et al., 2013; Hu X. et al., 2013). To mitigate the potential harm associated with exposure to sexual orientation, individuals who experience internalised dislike often choose to actively avoid emotional connections, leading to attachment avoidance (Guzmán-González et al., 2023). According to Self-Determination Theory, the failure to fulfill of an individual’s intrinsic psychological needs is detrimental to the development of their self-system (Ryan and Deci, 2024). Consequently, internalised dislike causes lesbians to suppress their psychological needs, which, in turn, undermines lesbians’ confidence in establishing intimate relationships. Therefore, it is hypothesized that internalised homophobia mediates the relationship between dark personality traits and adult attachment (H2).

### 2.3 The mediating role of self-esteem

Self-esteem refers to an individual’s overall assessment and feelings regarding their worth, abilities, and importance. Individuals with pronounced dark personality traits can be affected by pressure from minority groups, such as the social evaluation threat associated with homophobia, which can bypass their ‘overt narcissism’ defense mechanism. This occurs because internalised homophobia is seen as a threat to conforming to social norms, which activates their underlying sensitivity to ‘social exclusion’. This disruption can break down their ‘inflated self-esteem’ defense, revealing their ‘unstable self-worth’ and ultimately resulting in lower self-esteem (Skobkareva, 2020; Yin et al., 2025). Studies have shown that those with high dark personality traits tend to have lower self-esteem and exhibit high levels of neuroticism, anxiety, and susceptibility to depression (Black, 2013; Čopková, 2023).

Acceptance of one’s sexual orientation, as commonly referred to a strong sense of self-worth. Individuals with low self-esteem may conceal their identity due to fear of rejection, resulting in internal conflict and worsening insecure attachments (Veneziani et al., 2024). Homosexuals with high self-esteem are more likely to actively advocate for equal rights and reject unfair treatment, whereas those with low self-esteem may become trapped in a cycle of ‘self-depreciation → passive acceptance of discrimination’. Murray et al. (2015) found that individuals with low self-esteem were more prone to interpret their partner’s brief moments of coldness as a ‘rejection signal’ in intimate relationships, which can trigger anxiety or avoidance behaviors. Those with low self-esteem often resort to inhibitory strategies (e.g., repressing emotions, and avoiding communication), which further intensified attachment avoidance (He et al., 2010). In terms of neural mechanisms, fMRI studies have found that low self-esteem respond to social rejection with reduced activation in the prefrontal cortex (responsible for rational regulation) and enhanced activation in the amygdala (emotional response), exacerbating anxiety responses (Nakao et al., 2011). Therefore, the hypothesis 3 is that self-esteem mediates the relationship between dark personality traits and adult attachment (H3).

### 2.4 The chain mediation of internalised homophobia and self-esteem

The Psychological Mediators of Minority Stress (PMSM) model posits that the mental health challenges faced by sexual minorities stem from both external stress (discrimination, violence) and internal stress (internalised stigma, rejection of expectations), with internalised homophobia serving as a the central mediator, which leads directly to difficulties in self-esteem and interpersonal relationship (Li et al., 2023). Identity development models emphasise that individuals typically navigate a process of identity confusion → identity acceptance → identity integration. However dark personality traits may impede this process (e.g., narcissists are more likely to deny their true selves in order to maintain a sense of ‘superiority’), exacerbate internalised homophobia, diminish self-esteem, and undermine attachment security (Balasubramaniam and Alex, 2024). Therefore, the hypothesis 4 is advanced that internalised homophobia and self-esteem are chain-mediated between dark personality traits and adult attachment (H4).

### 2.5 Hypothesis of the current study

This study examined the relationship between dark personality traits and adult attachment, and verified the pathways in which internalised homophobia and self-esteem play a role. Specifically, the following four hypotheses were formulated:

H1: Dark personality traits co-influences adult attachment in lesbians; H2: Internalised homophobia mediates the relationship between dark personality traits and adult attachment; H3: Self-esteem mediates the relationship between dark personality traits and adult attachment; H4: Internalised homophobia and self-esteem are chain-mediated between dark personality traits and adult attachment.

**Figure 1:**
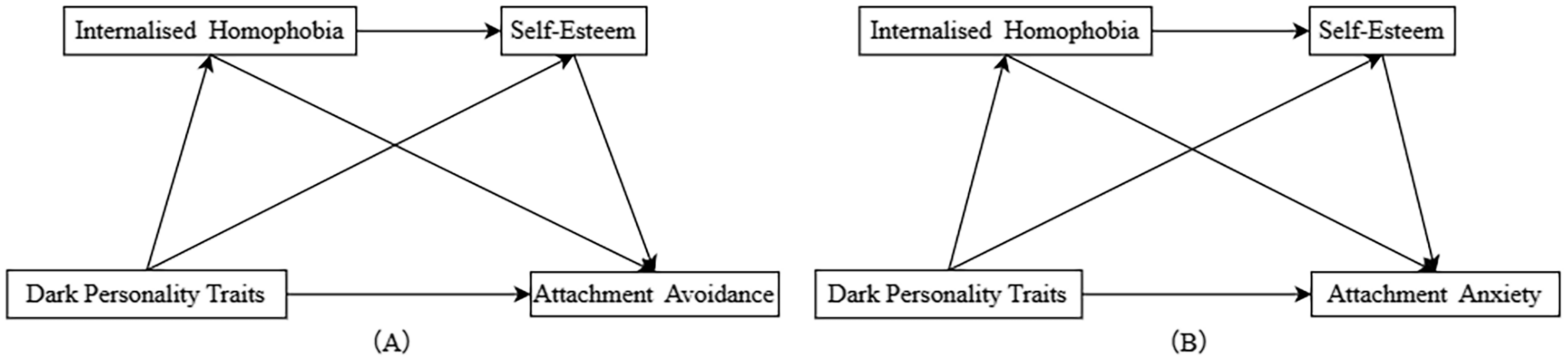
Research framework diagram.

The research framework diagram is shown in Figure 1.

## 3 Materials and methods

The study was approved by the Medical Ethics Committee of North China University of Science and Technology in accordance with the Declaration of Helsinki. Each participant read the informed consent form and agreed to participate in the study.

### 3.1 Participants

This study took a convenience and snowball sampling, with participants were recruited anonymously via a web-based survey disseminated through multiple channels: (a) The L, a lesbian-specific dating platform; (b) lesbian-affiliated QQ groups; and (c) mainstream Chinese social media platforms (Xiaohongshu, Douyin, and Weibo). Based on the definition by Meyer and Wilson’s (2009), this study included individuals who psychologically identify with their homosexual orientation. Considering that the sexual orientation of the underage group is still unstable (Spigarelli, 2007), inclusion criteria were limited to lesbian individuals over 18 years of age who self-identified as lesbians, had no major mental illnesses, and had not received professional psychological interventions or treatments in the past 3 months.

The formula $n=Z_{\alpha /2}^{2}\times [P(1-P)]/\delta^{2}$ was used to estimate the sample size (Huang, 2015), where *n* was the sample size, $Z_{\alpha /2}$ was the critical value of the score for the two-sided test α=0.05, δ was the permissible error, and Pwas the probability value. According to previous studies, the rate of social media use among the lesbian community is about 80% (Chan, 2023). In this study, the minimum sample size was calculated to be 307 with a 95% confidence interval ($Z_{\alpha /2}=1.96\approx 2$, δ=0.05, P=80%), after accounting for a 20% uplift for incomplete data, missing samples, and attention to screening. Between late March and mid-April 2025, a total of 1,188 questionnaires were collected with 1,136 valid questionnaires, yielding a valid response rate of 95.62%. The participants’ age range was 18–40 years (*M* = 21.57, SD = 3.716). Regarding education level, 933 (82.13%) had a bachelor’s degree or higher, 163 (14.35%) had a college degree, 35 (3.08%) had secondary or high school education, and 5 (0.44%) had junior high school or lower. Of the participants, 524 (46.13%) were only children, while 612 (53.87%) were not.

### 3.2 Tool

#### 3.2.1 Dirty dozen (DD)

Dark personality traits was measured using the Chinese version of the revised Dark Dozen (DD) (Geng et al., 2015). The scale includeds three dimensions: machiavellianism (e.g., ‘I tend to manipulate others to achieve my own ends’), psychopathy (e.g., ‘I am cold and numb’), and narcissism (e.g., ‘I want others to praise me’). Respondents were asked to rate 12 items (4 items for each dimention) on a seven-point Likert scale, ranging from 1 to 7. A total score was calculated across all items, with higher scores indicating higher levels of dark personality traits. The Cronbach’s alpha coefficient for this scale in the present study was 0.805 (0.797 for the Machiavellianism subscale, 0.642 for the Psychopathy subscale, and 0.809 for the Narcissism subscale).

#### 3.2.2 Internlised homophobia scale (IHS)

Internalised homophobia was measured using the Chinese version of the revised Internalised Homophobia Scale (IHS) (Li et al., 2014). The scale consists of 8 items, with respondents asked to rate each item on a five-point Likert scale ranging from 1 to 5. A total score was calculated across all items, with higher scores indicating higher levels of internalised homophobia.

The scale was originally designed for gay men. Therefore, the wording of items was modified to more adequately encompass lesbian experiences. Gender-specific expressions relevant only to gay men were removed. Examples include changing ‘I have tried to make myself less attracted to men’ to ‘I have tried to make myself less attracted to women’, changing ‘I wish I could feel more sexually attracted to women’ to ‘I wish I could feel more sexually attracted to men’, and changing ‘I have tried to make myself more sexually attractive to women’ to ‘I have tried to make myself more sexually attractive to men’.

A small-scale pilot test was conducted with 150 lesbian participants prior to the main study. The results indicate that the revised scale is well suited to the lesbian population, with item-total correlation coefficients ranging from 0.520 to 0.795 (Supplementary Table S1). The revised scale demonstrated good internal consistency in the initial test (Cronbach’s alpha coefficient of 0.765). In the formal test, this coefficient further improved to 0.792.

#### 3.2.3 Rosenberg self-esteem scale (RSES)

Self-esteem was measured using the Rosenberg Self-Esteem Scale (RSES) (Wang et al., 1999). This scale assesses individuals’ overall level of self-esteem, without focusing on specific qualities or characteristics. The scale consists of 10 items, with respondents rating each item on a four-point Likert scale ranging from 1 to 4. A total score was calculated across all items, with higher scores indicating higher levels of self-esteem. The Cronbach’s alpha coefficient for this scale in the present study was 0.828.

#### 3.2.4 Experiences in close relationships inventory (ECR)

Adult attachment style was measured using the Chinese version of the revised the Experiences in Close Relationships Inventory (ECR) (Li and Kato, 2006). The scale comprises two dimensions: attachment avoidance (e.g., ‘I try to avoid becoming too close to my lover’) and attachment anxiety (e.g., ‘I worry that I will be abandoned’). Respondents were asked to rate 36 items (18 items each for attachment avoidance and attachment anxiety) on a seven-point Likert scale ranging from 1 to 7. Total scores for each dimension were calculated to obtain composite scores for attachment avoidance and attachment anxiety, respectively. Higher attachment anxiety scores indicate a greater tendency to feel anxious and insecure in intimate relationships, while higher attachment avoidance scores reflect a greater tendency to be avoidant and independent in intimate relationships. The Cronbach’s alpha coefficient for the scales in this study was 0.876 (0.883 for the Attachment Avoidance subscale and 0.903 for the Attachment Anxiety subscale).

### 3.3 Statistical methods

Statistical analyses were conducted using SPSS 27.0. Pearson correlation analysis was performed to examine the relationships between variables. Model 6 of the SPSS macro program PROCESS developed by Hayes (2012), was used to test for the chain mediation of Internalised homophobia and self-esteem in the relationship between dark personality traits and adult attachment. The significance of the mediating effect was assessed using 5,000 bootstrap samples with bias correction. A 95% confidence interval (CI) that does not contain zero indicates a significant mediating effect (Wen et al., 2010).

## 4 Result

### 4.1 Common method bias test

Common method bias was assessed using the Harman’s one-factor test. The results identified 13 factors with eigenvalues greater than 1 without rotation. The first factor accounted for 16.554% of the variance, well below the 40% threshold (Yan et al., 2020), indicating that common method bias was not a significant concern in this study.

### 4.2 Correlation analysis of variables

Pearson correlation analyses revealed that dark personality traits was significantly positively correlated with internalised homophobia, attachment avoidance and attachment anxiety, and significantly negatively correlated with self-esteem (*r* = 0.136, *p* < 0.01; *r* = 0.130, *p* < 0.01; *r* = 0.348, *p* < 0,01; *r* = −0.112, *p* < 0.01); Internalised homophobia was significantly positively correlated with attachment avoidance and attachment anxiety, and significantly negatively correlated with self-esteem (*r* = 0.243, *p* < 0.01; *r* = 0.175, *p* < 0.01; *r* = −0.178, *p* < 0.01); Self-esteem was significantly negatively correlated with attachment avoidance and attachment anxiety (*r* = −0.329, *p* < 0.01; *r* = −0.371, *p* < 0.01). Means, standard deviations, and correlations across all variables are presented in Table 1.

**Table 1:** Correlation between variables.

Variables	1	2	3	4	5
1. Dark personality traits	1				
2. Internalised homophobia	0.136**	1			
3. Self-esteem	−0.112**	−0.178**	1		
4. Attachment avoidance	0.130**	0.243**	−0.329**	1	
5. Attachment anxiety	0.348**	0.175**	−0.371**	0.46	1
*M*	42.95	11.59	29.34	55.92	78.05
SD	11.257	4.284	4.990	17.861	19.199

*N* = 1,136, **p < 0.01.

### 4.3 Analysis of intermediation effects

Before constructing the chained mediation model, to ensure that the measurement data conformed to a normal distribution, normality tests were conducted using SPSS 27.0, and the skewness and kurtosis values of each variable were calculated. The results showed that variables such as dark personality traits, internalised homophobia, self-esteem, and attachment anxiety conformed to a normal distribution, while attachment avoidance did not significantly deviate from a normal distribution (Supplementary Table S2) (Kline, 2023). The sample data can be used directly for subsequent analysis.

**Figure 2:**
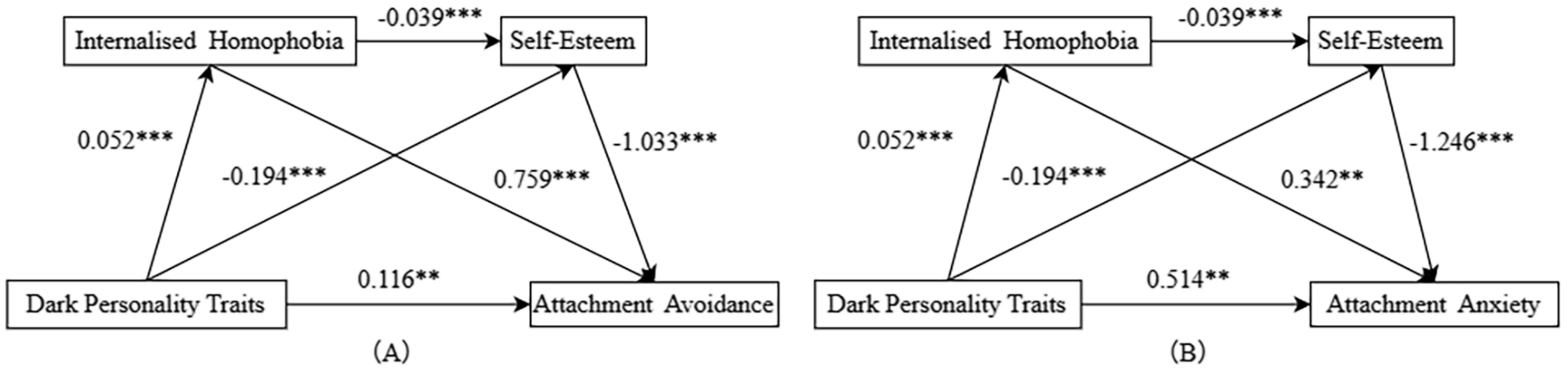
Path diagram of the chained mediating model. **p < 0.01, ***p< 0.001.

A chain mediation model was tested using Hayes’ PROCESS macro (Model 6) to examine the hypothesized pathways (Figure 2). As a saturated model (df = 0) with an equal number of estimated parameters and covariance matrix elements, fit indices were not applicable according to structural equation modeling conventions (Steeger and Gondoli, 2013). Path analyses (see Table 2) revealed the following that: (a) Dark personality traits co-influenced internalised homophobia (*β* = 0.052, *p* < 0.001), attachment avoidance (*β* = 0.514, *p* < 0.01) and attachment anxiety (*β* = 0.116, *p* < 0.01), while reversely impacting self-esteem (*β* = −0.194, *p* < 0.001); (b) Internalised homophobia negatively influenced self-esteem (*β* = −0.039, *p* < 0.001), while co-influenced attachment avoidance (*β* = 0.759, *p* < 0.001) and attachment anxiety (*β* = 0.342, *p* < 0.01); (c) Self-esteem had an inverse effect on attachment avoidance (*β* = −1.033, *p* < 0.001) and attachment anxiety (*β* = −1.246, *p* < 0.001).

**Table 2:** Results of the chain mediation model.

Result variables	Predictive variables	*R*	*R* ^2^	*F*	*β*	*t*	95% CI
M1	X	0.136	0.018	21.296***	0.136	4.615***	[0.029, 0.074]
M2	X	0.199	0.039	23.437***	−0.089	−3.053**	[−0.065, −0.014]
M2	M1				−0.166	−5.657***	[−0.261, −0.127]
Y1	X	0.386	0.148	66.002***	0.073	2.629**	[0.029, 0.202]
Y1	M1				0.182	6.484***	[0.529, 0.988]
Y1	M2				−0.289	−10.317***	[−1.229, −0.837]
Y2	X	0.489	0.239	118.301***	0.302	11.476***	[0.427, 0.602]
Y2	M1				0.076	2.872**	[0.108, 0.575]
Y2	M2				−0.324	−12.239***	[−1.446, −1.046]

X, dark personality traits; M1, internalised homophobia; M2, self-esteem; Y1, attachment Avoidance; Y2, attachment anxiety; Coefficients are standardized; Bootstrap samples = 5,000; CI, confidence interval; **p < 0.01, ***p < 0.001.

Mediation effect analyses (see Table 3) revealed that the internalised homophobia mediated the relationship between dark personality traits and attachment avoidance [*β =* 0.039, 95% CI (0.016, 0.067)] and attachment anxiety [*β =* 0.018, 95% CI (0.005, 0.035)]. Self-esteem mediated the relationship between dark personality traits and attachment avoidance [*β =* 0.041, 95% CI (0.012, 0.073)] and attachment anxiety [*β =* 0.049, 95% CI (0.032, 0.087)]. Additionally, the interlocking mediation effect between internalised homophobia and self-esteem was also significant [*β =* 0.010, 95% CI (0.004, 0.017); *β =* 0.013, 95% CI (0.005, 0.020)]. The present study confirms that the lesbian dark personality traits is a significant positive factor of their insecure attachment, operating through three main pathways: (a) dark personality traits → internalised homophobia → attachment avoidance and attachment anxiety; (b) dark personality traits → self-esteem → attachment avoidance, and attachment anxiety; (c) dark personality traits → internalised homophobia → self-esteem → attachment avoidance and attachment anxiety.

**Table 3:** Decomposition of mediating, direct and total effects.

Model pathway	*β*	*SE*	95% CI		Percent (%)
			LLCL	ULCL	
Total effect					
X → Y1	0.207	0.047	0.115	0.298	100
X → Y2	0.594	0.048	0.501	0.687	100
Direct effect					
X → Y1	0.116	0.044	0.029	0.203	56.099
X → Y2	0.514	0.045	0.427	0.603	86.556
Indirect effect					
X → M1 → Y1	0.039	0.013	0.016	0.067	15.924
X → M2 → Y1	0.041	0.016	0.012	0.073	19.894
X → M1 → M2 → Y1	0.010	0.003	0.005	0.017	4.985
X → M1 → Y2	0.018	0.008	0.005	0.035	2.976
X → M2 → Y2	0.049	0.018	0.014	0.087	8.349
X → M1 → M2 → Y2	0.012	0.004	0.005	0.021	2.104

X, dark personality traits; M1, internalised homophobia; M2, self-esteem; Y1, attachment avoidance; Y2, attachment anxiety; Bootstrap samples = 5,000; *β*, path coefficient; SE, standard error; CI, confidence interval; LLCL, lower limits confidence interval; ULCL, upper limits confidence interval.

Overall, the total effect of dark personality traits on attachment avoidance was 0.207, with 56.15% (0.116) of this effect being direct and 43.85% (0.091) being indirect. The total effect of dark personality traits on attachment anxiety was 0.594, with 86.58% (0.514) of this effect being direct and 13.42% (0.079) being indirect.

## 5 Discussion

### 5.1 Theoretical implications

This study constructed a theoretical framework integrating lesbian dark personality traits, internalised homophobia, self-esteem and adult attachment based on the Minority Stress Mediation Model and Well-being Goal Theory. The present findings reveal a significant positive association between dark triad traits and adult attachment insecurity within lesbian populations, extending prior evidence from heterosexual samples (Brewer et al., 2018; Cai et al., 2020; Nickisch et al., 2020; Bloxsom et al., 2021; Shirazi et al., 2024) to sexual minority contexts. Previous research has confirmed that attachment avoidance and attachment anxiety to the development of persistent dark personality triads, which tend to exhibit antisocial traits over time (Bowlby, 1982; Amiri and Jamali, 2019; Fard et al., 2022). The present study, in turn, confirms the predictive role of dark triad traits on insecure attachment (Brewer et al., 2018; Cai et al., 2020), which suggests that dark personality traits and adult attachment are dynamic influences that interact in both directions, consistent with a lifelong developmental perspective.

The results of the present study indicate that internalised homophobia mediates the relationship between dark personality traits and adult attachment, suggesting that internalised homophobia is positively correlated with dark personality traits and consequently, influences attachment patterns. This may be because lesbian women with lower levels of internalised homophobia tend to report higher subjective well-being (Fang et al., 2014; Lorenzi et al., 2015; Garro et al., 2022), and their positive views on their sexual orientation help reduce insecure attachment, which aligns with the findings of Keleher et al. (2010). Additionally, lesbian women with lower levels of internalised homophobia are better at utilising social support (Chard et al., 2015; Calvo et al., 2021; Anil and Raveendran, 2022), enabling them to better achieve personal goals and social needs, and exhibit more secure attachment patterns, consistent with the goal theory of happiness (Emmons and Kaiser, 1996).

The results of this study indicate that self-esteem mediates the relationship between dark personality traits and adult attachment. This suggests that dark personality traits influence attachment patterns by affecting an individual’s level of self-esteem, which in turn influences attachment patterns. This finding aligns with the results of previous studies. The fear management theory of self-esteem posits that self-esteem can help alleviate anxiety (Sowislo and Orth, 2013; Rossi et al., 2020; Rossi et al., 2024). Multiple studies have shown that individuals with lower self-esteem are more susceptible to social anxiety (Ahmad et al., 2013; Iancu et al., 2015). This is because, compared to those with low self-esteem, lesbian women with higher self-esteem generally possess a stronger sense of self-worth (Crocker et al., 2006; Isserow, 2023; Bırni and Eryılmaz, 2024). They are more willing to share their thoughts in social settings and actively participate in teamwork, thereby enhancing their social and interpersonal skills (Shetty et al., 2023), and developing more secure attachment patterns.

The results of the present study suggest that internalised homophobia and self-esteem play a significant chain-mediating role between lesbian dark personality traits and adult attachment. This study hypothesise that this relationship is primarily associated with the narcissistic dimension of dark personality traits. Grandiosity narcissists are characterized by perceived lack of empathy and impaired interpersonal relationships (Southard et al., 2015; Urbonaviciute and Hepper, 2020; di Giacomo et al., 2023). Lesbians face a series of practical issues including social exclusion, family conflucts, and whether or not to come out, demonstrate higher prevalence of vulnerable narcissism rather than grandiose narcissism (Akçiçek, 2019). The main characteristics include apparent self-consciousness and sensitivity, as well as a strong need for self-centredness (Lee et al., 2023; Jiang et al., 2024), easily damaged self-esteem, and a tendency to be defensive (Hart et al., 2017). Vulnerable lesbian narcissists are prone to internalising society’s negative attitudes towards homosexuality, forming internalised homophobia, which leads to self-denial and low self-esteem. Low self-esteem exacerbates attachment avoidance and attachment anxiety, causing them to fear abandonment and resist deep emotional connections in intimate relationships, ultimately leading to an insecure attachment pattern.

It is worth noting that existing research has shown that the attractiveness of dark personality traits may differ between heterosexual and lesbian relationships. In heterosexual contexts, Carter et al. (2014) found that men with higher levels of dark personality traits were more likely to be perceived as attractive by women. One possible explanation is that such men often exhibit stronger aggression, ambition, and leadership, traits that are traditionally less associated with women in gender role perceptions (Carli and Eagly, 2011; Hall, 2014; Tremmel and Wahl, 2023). However, it is important to note that while this study supports the aforementioned view, empirical research on the attractiveness of dark personality traits in lesbian relationships remains limited. Further studies are needed to validate and explore potential differences between these two relationship patterns.

### 5.2 Practical implications

First, the theoretical framework constructed in this study facilitates an understanding of the personality characteristics, role identity, and self-esteem levels of lesbians at the individual level, as well as conflict management styles and behavioral motivations at the relationship level. Research has shown that early experiences do not dictate outcomes in adulthood (Fraley and Roisman, 2019). For individuals who have faced rejection and trauma, intimate relationships characterized by love and trust can offer a secure psychological working model (Davila et al., 1999). Therefore, this study suggests that lesbian women should fully recognise the covariation and developmental nature of personality traits and attachment patterns. They should also recognise and cultivate positive personality traits, appropriately manage their ‘bright’ and ‘dark’ personalities, and proactively improve their attachment patterns.

Secondly, this study suggests that in clinical practice, in addition to screening for personality disorders, it is also essential to systematically assess clients’ attachment styles. If highly avoidant and anxious attachment styles, as well as pathological personalities can be identified, counsellors will be able to gain a deeper understanding of clients’ traumatic experiences and their functional impairments in growth and interpersonal relationships. This focus counselling on specific goals such as relationship stability, emotional regulation, and identity integration.

In addition, in lesbian counselling, counsellors should work to affirm the identity of lesbians and focus on reducing their internalised homophobia. For those with high internalised homophobia, they should focus on fostering self-confidence in various aspects, avoiding their tendency to use sexual orientation as the sole criterion for self-evaluation, and promoting their overall self-esteem. In addition, they should be encouraged to develop diverse interpersonal resources so that they can have meaningful exchanges with their peers and alleviate their feelings loneliness, thereby enhancing their subjective well-being (Fang et al., 2014; Lorenzi et al., 2015; Garro et al., 2022). However, the effectiveness of reducing insecure attachment by alleviating internalised self-disgust has been found to be temporary (Sherry, 2007), which may be related to the fact that Chinese lesbian women have long been exposed to a collectivist cultural context and face pressures from both family and career (Hu J. C. et al., 2013; Hu X. et al., 2013; Shao et al., 2018). Therefore, enhancing the identity recognition of Chinese lesbian women and addressing internal self-loathing must be pursued consistently and over the long term.

Finally, in view of the fact that more and more legislators begin to recognize the rights and interests of sexual minorities, it is also necessary to popularise, clarify, and discuss lesbianism as a sexual minority in a timely manner at the level of policymaking and mass communication, so as to broaden the scope of protection for this community.

### 5.3 Limitations and suggestions for future research

There are certain limitations and shortcomings of this study, which are as follows:

This study employed convenience and snowball sampling, which facilitates access to the lesbian population for research purposes, while these methods have significant limitations. These sampling methods are prone to self-selection bias, with samples primarily comprising younger, highly educated individuals, making it difficult to represent lesbians from diverse age groups, educational backgrounds, and socioeconomic statuses. Additionally, due to the constraints of the Chinese cultural context, the generalisability of the research findings to lesbian populations in other cultural contexts is questionable. Future studies could employ stratified or random sampling methods to include lesbian women from various age groups, educational levels, and regions (encompassing diverse cultural contexts). The scope could also be expanded to include transgender and bisexual indivuduals to enhance sample representativeness and test the applicability of research findings across a broader range of sexual minority groups.

This study did not include covariates such as age or educational attainment. The snowball sampling method used in this study collected samples that were highly homogeneous, thereby limiting the variability of demographic factors to a certain extent and partially mitigating their potential impact on the dependent variable. Furthermore, the mediational model structure in this study is already quite complex. Adding multiple covariates would increase its complexity, reduce model interpretability and potentially decrease statistical power due to reduced degrees of freedom. To preserve model robustness and clarity, covariates were excluded. However, as internalised homophobia and self-esteem may be influenced by sociodemographic factors, failing to control for them may reduce the accuracy of the results. Future research should further optimise model design by incorporating covariates such as age and educational attainment to control for potential confounding factors and improve model accuracy and precision. Additionally, the Cronbach’s alpha for the psychopathy subscale in the “Dirty Dozen” is only 0.642, below the acceptable standard (0.7), which may result in a discrepancy between the psychopathy traits measured by the subscale and reality. Since this subscale is used to measure stable traits in dark personality, such as low empathy and interpersonal coldness, insufficient reliability may weaken the association between dark personality and other variables (such as internalised homophobia). When combined with the reliable results of other subscales, the overall model still shows significant effects, indicating that the core conclusions remain credible despite reliability limitations. This suggests that future research should replace or supplement unreliable measurement tools or adopt more mature “dark traits” measurement tools to improve data quality.

Since this study is a cross-sectional study, it is not possible to infer causal relationships between dark personality traits, internalised homophobia, self-esteem and adult attachment, nor the temporal trends of these factors. Future research could employ longitudinal tracking designs (such as cross-lagged models) to observe the dynamic changes in variables such as dark personality traits, internalised homophobia, self-esteem, and attachment patterns over the long term, thereby clarifying the causal sequence among these variables. Additionally, further theoretical integration could distinguish between the ‘overt-covert’ dimensions of narcissism, combine minority stress theory, and analyse mechanisms such as ‘social isolation’ and ‘stress eroding self-worth’ thereby enhancing the theoretical contributions of the research. All findings in this study are based on participants’ self-reports. Whereas measures of dark personality, internalised dislike, and insecure attachment would involve some of the negative or negative behaviors. Although the present study used a completely anonymous online survey, it may still be influenced by social approbability, leading to biased results. Subsequent studies may appropriately add behavioral observations or behavioral experiments to validate the findings.

## 6 Conclusion

This study highlights the critical role of dark personality traits in improving adult attachment patterns among lesbian women through the mediating effects of internalised homophobia and self-esteem. Drawing on the minority stress mediation model and the theory of happiness goals, it confirms the covariation and dynamic development between dark personality traits and adult attachment patterns. Lesbian women with lower levels of dark personality traits exhibit lower levels of internalised homophobia and higher levels of self-esteem, enabling them to better adapt to social rejection and social pressure, thereby forming relatively secure and healthy attachment patterns. These findings provide valuable insights for counselling and clinical practice targeting lesbian women. Future research should explore the differences in attachment patterns among a broader lesbian population and in different cultural contexts to further enrich our understanding of how these concepts deeply influence lesbians’ intimate relationships.

## Data Availability

The original contributions presented in the study are included in the article/Supplementary material, further inquiries can be directed to the corresponding author.
