# Supplementary material for: Mechanisms of dark personality traits in lesbian adult attachment: a chain-mediated model
Source: Front Psychol. 2025 Jul 23;16:1619432. doi: 10.3389/fpsyg.2025.1619432 (PMC12326747; doi:10.3389/fpsyg.2025.1619432)
Supplement: Supplementary file 1 [file Supplementary_file_1.docx]

**Supplementary Materials**

Table S1 Pr**e-Tes**t Results of IHS’s Item-Total Correlation

|  | IHS_1 | IHS_2 | IHS_3 | IHS_4 | IHS_5 | IHS_6 | IHS_7 | IHS_8 |
| --- | --- | --- | --- | --- | --- | --- | --- | --- |
| IHS | 0.599** | 0.795** | 0.679** | 0.705** | 0.745** | 0.589** | 0.739** | 0.520** |

Note: N=150, $**P<0.01$, IHS indicates the total score on the scale. IHS_1 represents the first item on the IHS, IHS_2 represents the second item on the IHS, and so on.

Table S2 Results of the Normality Test

| Variables | Skewness | | Kurtosis | |
| --- | --- | --- | --- | --- |
|  | Value | SE | Value | SE |
| 1. Dark Personality traits | 0.251 | 0.072 | -0.116 | 0.145 |
| 1. Internalized | 2.070 | 0.072 | 6.564 | 0.145 |
| 1. Self-Esteem | -0.095 | 0.072 | -0.258 | 0.145 |
| 1. Attachment Avoidance | 0.336 | 0.072 | -0.431 | 0.145 |
| 1. Attachment Anxiety | -0.246 | 0.072 | -0.242 | 0.145 |

Note: Skewness should be between ±3; Kurtosis should be between ±10.
